# Supplementary material for: The bile acid metabolome in umbilical cord blood and meconium of healthy newborns: distinct characteristics and implications
Source: PeerJ. 2024 Dec 13;12:e18506. doi: 10.7717/peerj.18506 (PMC11648689; doi:10.7717/peerj.18506)
Supplement: Supplemental Information 3 [file peerj-12-18506-s003.docx]

|  | primary bile acids | secondary bile acids | unconjugated bile acids | conjugated bile acids | T-conjugated bile acids | G-conjugated bile acids |
| --- | --- | --- | --- | --- | --- | --- |
| umbilical cord blood (nmol/L) | 2074.20  (1508.03, 2969.55) | 772.18  (580.64, 892.19) | 325.81  (222.87, 438.76) | 2375.99  (1956.97, 3740.23) | 1583.11  (1235.97, 2389.81) | 820.63  (587.42, 1350.42) |
| Meconium  (nmol/g) | 2825.39  (1472.38, 5065.02) | 3093.78  (2223.83, 4048.72) | 118.12  (44.64, 171.40) | 5292.88  (4312.29, 7719.61) | 4527.50  (3692.15, 6622.30) | 731.62  (584.60, 986.82) |
