# Supplementary material for: The bile acid metabolome in umbilical cord blood and meconium of healthy newborns: distinct characteristics and implications
Source: PeerJ. 2024 Dec 13;12:e18506. doi: 10.7717/peerj.18506 (PMC11648689; doi:10.7717/peerj.18506)
Supplement: Supplemental Information 4 [file peerj-12-18506-s004.docx]

|  | umbilical cord blood | meconium | Z | *P* |
| --- | --- | --- | --- | --- |
| primary bile acids/secondary bile acids | 2.64 (2.49, 5.70) | 0.99 (0.37, 1.58) | -4.54 | < 0.001 |
| unconjugated bile acids/conjugated bile acids | 0.14 (0.07, 0.18) | 0.01 (0.01, 0.04) | -3.88 | < 0.001 |
