# Supplementary material for: The bile acid metabolome in umbilical cord blood and meconium of healthy newborns: distinct characteristics and implications
Source: PeerJ. 2024 Dec 13;12:e18506. doi: 10.7717/peerj.18506 (PMC11648689; doi:10.7717/peerj.18506)
Supplement: Supplemental Information 5 — Horizontal coordinate is meconium, vertical coordinate is umbilical cord blood. [file peerj-12-18506-s005.docx]

|  |  | CA | TCA | GCA | CDCA | TCDCA | GCDCA |
| --- | --- | --- | --- | --- | --- | --- | --- |
| CA | r | 0.39 | 0.10 | 0.37 | 0.02 | 0.01 | 0.02 |
|  | *P* | 0.15 | 0.71 | 0.17 | 0.94 | 0.97 | 0.95 |
| TCA | r | -0.21 | 0.12 | 0.10 | -0.33 | -0.02 | -0.10 |
|  | *P* | 0.45 | 0.68 | 0.72 | 0.23 | 0.94 | 0.71 |
| GCA | r | 0.01 | -0.15 | -0.01 | -0.13 | -0.19 | -0.11 |
|  | *P* | 0.98 | 0.60 | 0.98 | 0.66 | 0.51 | 0.69 |
| CDCA | r | 0.09 | 0.14 | 0.16 | -0.05 | 0.11 | -0.01 |
|  | *P* | 0.76 | 0.61 | 0.57 | 0.87 | 0.69 | 0.98 |
| TCDCA | r | -0.11 | 0.33 | 0.08 | 0.03 | 0.41 | 0.25 |
|  | *P* | 0.69 | 0.23 | 0.77 | 0.91 | 0.13 | 0.38 |
| GCDCA | r | -0.02 | 0.46 | 0.24 | 0.07 | 0.43 | 0.26 |
|  | *P* | 0.94 | 0.08 | 0.39 | 0.81 | 0.11 | 0.35 |
| GCDCA-3S | r | 0.12 | 0.15 | 0.24 | 0.34 | 0.19 | 0.38 |
|  | *P* | 0.68 | 0.59 | 0.38 | 0.22 | 0.49 | 0.16 |
| GCDCA-3Glu | r | -0.08 | 0.25 | 0.16 | 0.09 | 0.51 | 0.42 |
|  | *P* | 0.77 | 0.36 | 0.58 | 0.76 | 0.05 | 0.12 |
