# Supplementary material for: The bile acid metabolome in umbilical cord blood and meconium of healthy newborns: distinct characteristics and implications
Source: PeerJ. 2024 Dec 13;12:e18506. doi: 10.7717/peerj.18506 (PMC11648689; doi:10.7717/peerj.18506)
Supplement: Supplemental Information 6 — *, P < 0.05; **, P < 0.01; (−), Non-downstream secondary bile acids [file peerj-12-18506-s006.docx]

|  |  | CA | TCA | GCA | CDCA | TCDCA | GCDCA | GCDCA-3S | GCDCA-3Glu |
| --- | --- | --- | --- | --- | --- | --- | --- | --- | --- |
| LCA | r | 0.29 | 0.18 | 0.44 | -0.19 | -0.22 | -0.08 | 0.11 | -0.40 |
|  | *P* | 0.29 | 0.52 | 0.10 | 0.49 | 0.43 | 0.78 | 0.69 | 0.14 |
| isoalloLCA | r | -0.45 | -0.08 | -0.26 | -0.14 | -0.21 | -0.22 | -0.24 | 0.12 |
|  | *P* | 0.10 | 0.78 | 0.36 | 0.61 | 0.45 | 0.44 | 0.38 | 0.66 |
| isoLCA | r | 0.03 | -0.11 | 0.07 | 0.30 | -0.21 | -0.18 | -0.41 | -0.39 |
|  | *P* | 0.93 | 0.69 | 0.81 | 0.27 | 0.45 | 0.52 | 0.13 | 0.15 |
| TLCA | r | 0.21 | 0.05 | 0.13 | -0.21 | -0.02 | 0.21 | 0.19 | 0.23 |
|  | *P* | 0.44 | 0.85 | 0.66 | 0.45 | 0.94 | 0.46 | 0.49 | 0.41 |
| LCA-3S | r | 0.16 | -0.05 | 0.03 | -0.16 | -0.25 | 0.09 | -0.10 | -0.34 |
|  | *P* | 0.57 | 0.86 | 0.93 | 0.57 | 0.36 | 0.75 | 0.73 | 0.22 |
| 7-KetoLCA | r | 0.36 | -0.27 | -0.03 | -0.12 | -0.38 | -0.25 | 0.03 | 0.02 |
|  | *P* | 0.19 | 0.33 | 0.92 | 0.67 | 0.16 | 0.37 | 0.92 | 0.95 |
| 12-KetoLCA | r | 0.14 | 0.23 | 0.34 | -0.26 | -0.42 | -0.05 | 0.00 | -0.45 |
|  | *P* | 0.62 | 0.41 | 0.22 | 0.36 | 0.12 | 0.87 | 1.00 | 0.09 |
| GLCA-3S | r | -0.05 | -0.09 | 0.03 | -0.03 | 0.28 | 0.48 | 0.19 | -0.10 |
|  | *P* | 0.86 | 0.75 | 0.93 | 0.93 | 0.32 | 0.07 | 0.51 | 0.71 |
| DCA | r | 0.16 | -0.14 | 0.11 | -0.40 | **-0.61*^, (-)^** | -0.40 | -0.06 | -0.18 |
|  | *P* | 0.58 | 0.61 | 0.69 | 0.14 | **0.02** | 0.14 | 0.84 | 0.52 |
| TDCA | r | 0.23 | 0.12 | 0.02 | -0.14 | 0.13 | 0.30 | -0.09 | 0.11 |
|  | *P* | 0.41 | 0.68 | 0.95 | 0.62 | 0.64 | 0.28 | 0.76 | 0.69 |
| GDCA | r | 0.12 | -0.04 | 0.13 | -0.39 | -0.24 | 0.02 | 0.12 | 0.02 |
|  | *P* | 0.67 | 0.89 | 0.66 | 0.15 | 0.38 | 0.95 | 0.68 | 0.94 |
| DCA-3S | r | 0.20 | 0.00 | 0.16 | -0.21 | -0.45 | -0.21 | -0.30 | -0.46 |
|  | *P* | 0.48 | 0.99 | 0.58 | 0.44 | 0.10 | 0.44 | 0.27 | 0.08 |
| TωMCA | r | -0.15 | -0.17 | -0.41 | 0.46 | 0.44 | **0.52*** | -0.03 | 0.23 |
|  | *P* | 0.60 | 0.54 | 0.13 | 0.09 | 0.10 | **0.05** | 0.92 | 0.41 |
| TαMCA | r | 0.23 | 0.13 | 0.15 | 0.18 | 0.19 | 0.38 | -0.01 | -0.13 |
|  | *P* | 0.41 | 0.66 | 0.60 | 0.53 | 0.50 | 0.17 | 0.97 | 0.66 |
| αMCA | r | -0.04 | -0.02 | -0.08 | 0.15 | -0.19 | -0.19 | -0.16 | -0.26 |
|  | *P* | 0.89 | 0.95 | 0.77 | 0.59 | 0.51 | 0.51 | 0.58 | 0.34 |
| βMCA | r | -0.14 | -0.24 | -0.06 | 0.11 | -0.24 | -0.17 | -0.02 | -0.42 |
|  | *P* | 0.62 | 0.40 | 0.82 | 0.69 | 0.39 | 0.54 | 0.94 | 0.12 |
| HCA | r | -0.21 | **-0.61*^, (-)^** | -0.37 | -0.16 | -0.50 | -0.44 | -0.25 | -0.04 |
|  | *P* | 0.46 | **0.02** | 0.18 | 0.57 | 0.06 | 0.10 | 0.37 | 0.88 |
| THCA | r | -0.38 | -0.33 | **-0.65**^, (-)^** | 0.31 | 0.34 | 0.31 | -0.17 | 0.43 |
|  | *P* | 0.17 | 0.23 | **0.01** | 0.25 | 0.22 | 0.25 | 0.54 | 0.11 |
| GHCA | r | 0.00 | -0.23 | -0.42 | 0.41 | 0.03 | 0.20 | 0.00 | 0.19 |
|  | *P* | 0.99 | 0.41 | 0.12 | 0.13 | 0.91 | 0.48 | 0.99 | 0.49 |
| HDCA | r | 0.29 | 0.16 | 0.24 | -0.35 | **-0.66**** | -0.44 | -0.26 | -0.41 |
|  | *P* | 0.30 | 0.56 | 0.38 | 0.20 | **0.01** | 0.10 | 0.34 | 0.13 |
| GHDCA | r | -0.23 | -0.28 | -0.37 | -0.07 | 0.05 | 0.25 | 0.08 | 0.20 |
|  | *P* | 0.41 | 0.31 | 0.18 | 0.80 | 0.86 | 0.38 | 0.77 | 0.47 |
| βHDCA | r | 0.40 | 0.09 | 0.43 | -0.26 | -0.42 | -0.45 | -0.33 | -0.42 |
|  | *P* | 0.14 | 0.76 | 0.11 | 0.35 | 0.12 | 0.09 | 0.23 | 0.12 |
| THDCA | r | -0.03 | -0.15 | -0.31 | 0.24 | 0.50 | 0.44 | 0.03 | 0.31 |
|  | *P* | 0.91 | 0.60 | 0.26 | 0.38 | 0.06 | 0.10 | 0.91 | 0.26 |
| 7-DHCA | r | 0.04 | -0.05 | -0.03 | -0.07 | -0.43 | -0.23 | -0.31 | **-0.67**^, (-)^** |
|  | *P* | 0.88 | 0.85 | 0.93 | 0.80 | 0.11 | 0.41 | 0.27 | **0.01** |
| 12-DHCA | r | 0.30 | 0.03 | 0.17 | 0.19 | -0.02 | 0.01 | 0.20 | -0.46 |
|  | *P* | 0.28 | 0.92 | 0.55 | 0.50 | 0.95 | 0.98 | 0.47 | 0.09 |
| 3-DHCA | r | 0.11 | 0.02 | -0.03 | -0.13 | 0.02 | 0.18 | 0.38 | -0.09 |
|  | *P* | 0.70 | 0.95 | 0.93 | 0.64 | 0.94 | 0.52 | 0.17 | 0.74 |
| TUDCA | r | 0.08 | -0.04 | -0.31 | 0.33 | 0.39 | 0.41 | 0.03 | 0.40 |
|  | *P* | 0.79 | 0.88 | 0.26 | 0.24 | 0.16 | 0.13 | 0.91 | 0.14 |
| GUDCA | r | -0.07 | -0.23 | -0.25 | 0.21 | 0.34 | 0.43 | 0.38 | 0.31 |
|  | *P* | 0.81 | 0.41 | 0.36 | 0.44 | 0.22 | 0.11 | 0.16 | 0.26 |
| UDCA-7S | r | 0.27 | -0.07 | -0.01 | -0.03 | -0.48 | -0.28 | -0.45 | **-0.53*** |
|  | *P* | 0.33 | 0.81 | 0.97 | 0.92 | 0.07 | 0.31 | 0.09 | **0.04** |
