# Supplementary material for: The bile acid metabolome in umbilical cord blood and meconium of healthy newborns: distinct characteristics and implications
Source: PeerJ. 2024 Dec 13;12:e18506. doi: 10.7717/peerj.18506 (PMC11648689; doi:10.7717/peerj.18506)
Supplement: Supplemental Information 7 — *, P < 0.05; **, P < 0.01; (−), Non-downstream secondary bile acids [file peerj-12-18506-s007.docx]

|  |  | CA | TCA | GCA | CDCA | TCDCA | GCDCA |
| --- | --- | --- | --- | --- | --- | --- | --- |
| LCA | r | 0.51 | 0.21 | 0.38 | 0.21 | -0.01 | 0.09 |
|  | *P* | 0.05 | 0.45 | 0.16 | 0.46 | 0.99 | 0.74 |
| isoalloLCA | r | -0.15 | -0.13 | -0.16 | -0.03 | -0.11 | -0.01 |
|  | *P* | 0.60 | 0.64 | 0.57 | 0.92 | 0.69 | 0.98 |
| isoLCA | r | -0.16 | -0.29 | -0.33 | **-0.53*** | -0.40 | **-0.66**** |
|  | *P* | 0.58 | 0.30 | 0.23 | **0.04** | 0.14 | **0.01** |
| TLCA | r | **0.63*^, (-)^** | 0.48 | **0.57*^, (-)^** | **0.56*** | **0.58*** | **0.61*** |
|  | *P* | **0.01** | 0.07 | **0.03** | **0.03** | **0.02** | **0.02** |
| LCA-3S | r | 0.28 | 0.13 | 0.07 | -0.06 | -0.06 | -0.18 |
|  | *P* | 0.31 | 0.66 | 0.80 | 0.83 | 0.83 | 0.52 |
| 7-KetoLCA | r | **0.81**^, (-)^** | 0.30 | **0.58*^, (-)^** | **0.52*** | 0.28 | 0.46 |
|  | *P* | **0.00** | 0.27 | **0.02** | **0.05** | 0.31 | 0.09 |
| 12-KetoLCA | r | 0.36 | 0.11 | 0.24 | 0.16 | -0.09 | -0.03 |
|  | *P* | 0.18 | 0.69 | 0.38 | 0.58 | 0.75 | 0.93 |
| GLCA-3S | r | -0.11 | 0.02 | -0.23 | -0.28 | -0.02 | -0.20 |
|  | *P* | 0.70 | 0.95 | 0.41 | 0.32 | 0.95 | 0.48 |
| DCA | r | 0.42 | -0.12 | 0.18 | 0.20 | -0.24 | 0.03 |
|  | *P* | 0.12 | 0.67 | 0.52 | 0.48 | 0.38 | 0.91 |
| TDCA | r | 0.48 | **0.66**** | 0.48 | 0.14 | **0.57*^, (-)^** | 0.30 |
|  | *P* | 0.07 | **0.01** | 0.07 | 0.63 | **0.03** | 0.28 |
| GDCA | r | 0.48 | 0.19 | 0.30 | 0.32 | 0.18 | 0.44 |
|  | *P* | 0.07 | 0.50 | 0.27 | 0.25 | 0.53 | 0.10 |
| DCA-3S | r | 0.06 | -0.20 | -0.20 | -0.30 | -0.40 | -0.41 |
|  | *P* | 0.83 | 0.48 | 0.47 | 0.28 | 0.14 | 0.13 |
| TωMCA | r | 0.35 | **0.78**^, (-)^** | 0.49 | 0.05 | **0.65**** | 0.24 |
|  | *P* | 0.21 | **0.00** | 0.06 | 0.87 | **0.01** | 0.38 |
| TαMCA | r | 0.35 | 0.51 | 0.35 | -0.10 | 0.30 | 0.03 |
|  | *P* | 0.20 | 0.05 | 0.20 | 0.72 | 0.28 | 0.91 |
| αMCA | r | -0.04 | -0.15 | -0.03 | 0.30 | -0.03 | -0.02 |
|  | *P* | 0.89 | 0.59 | 0.93 | 0.27 | 0.91 | 0.94 |
| βMCA | r | 0.01 | -0.29 | -0.09 | 0.39 | -0.19 | -0.04 |
|  | *P* | 0.96 | 0.30 | 0.76 | 0.15 | 0.51 | 0.90 |
| HCA | r | 0.44 | -0.04 | 0.15 | 0.30 | -0.08 | 0.08 |
|  | *P* | 0.10 | 0.88 | 0.60 | 0.27 | 0.79 | 0.78 |
| THCA | r | 0.01 | 0.45 | 0.16 | -0.09 | 0.41 | 0.00 |
|  | *P* | 0.96 | 0.10 | 0.56 | 0.76 | 0.13 | 0.99 |
| GHCA | r | **0.58*^, (-)^** | **0.59*^, (-)^** | **0.63*^, (-)^** | 0.28 | 0.50 | 0.48 |
|  | *P* | **0.02** | **0.02** | **0.01** | 0.32 | 0.06 | 0.07 |
| HDCA | r | 0.41 | 0.08 | 0.31 | 0.07 | -0.23 | -0.15 |
|  | *P* | 0.13 | 0.79 | 0.26 | 0.81 | 0.42 | 0.59 |
| GHDCA | r | 0.51 | 0.50 | 0.44 | 0.49 | **0.55*** | **0.70**** |
|  | *P* | 0.05 | 0.06 | 0.10 | 0.06 | **0.04** | **0.00** |
| βHDCA | r | 0.19 | -0.20 | -0.12 | -0.27 | -0.37 | -0.42 |
|  | *P* | 0.49 | 0.47 | 0.68 | 0.33 | 0.17 | 0.12 |
| THDCA | r | 0.39 | **0.70**^, (-)^** | 0.44 | 0.25 | **0.74**** | 0.40 |
|  | *P* | 0.15 | **0.00** | 0.10 | 0.37 | **0.00** | 0.14 |
| 7-DHCA | r | 0.25 | -0.01 | 0.09 | 0.27 | -0.12 | -0.08 |
|  | *P* | 0.37 | 0.99 | 0.75 | 0.32 | 0.66 | 0.79 |
| 12-DHCA | r | 0.46 | 0.23 | 0.44 | **0.58*^, (-)^** | 0.15 | 0.21 |
|  | *P* | 0.09 | 0.40 | 0.10 | **0.02** | 0.59 | 0.45 |
| 3-DHCA | r | **0.71**** | **0.62*** | **0.76**** | **0.76**^, (-)^** | **0.52*^, (-)^** | **0.67**^, (-)^** |
|  | *P* | **0.00** | **0.01** | **0.00** | **0.00** | **0.05** | **0.01** |
| TUDCA | r | 0.45 | **0.78**^, (-)^** | **0.63*^, (-)^** | 0.37 | **0.84**** | **0.65**** |
|  | *P* | 0.09 | **0.00** | **0.01** | 0.18 | **0.00** | **0.01** |
| GUDCA | r | 0.36 | 0.38 | 0.38 | **0.60*** | **0.61*** | **0.78**** |
|  | *P* | 0.19 | 0.16 | 0.16 | **0.02** | **0.02** | **0.00** |
| UDCA-7S | r | 0.20 | -0.04 | -0.03 | -0.29 | -0.33 | -0.51 |
|  | *P* | 0.48 | 0.89 | 0.92 | 0.29 | 0.23 | 0.05 |
