# Supplementary material for: The bile acid metabolome in umbilical cord blood and meconium of healthy newborns: distinct characteristics and implications
Source: PeerJ. 2024 Dec 13;12:e18506. doi: 10.7717/peerj.18506 (PMC11648689; doi:10.7717/peerj.18506)
Supplement: Supplemental Information 8 — **, P < 0.01 [file peerj-12-18506-s008.docx]

| secondary bile acids shared by meconium and umbilical cord blood | r | *P* |
| --- | --- | --- |
| TαMCA | 0.11 | 0.69 |
| THCA | -0.08 | 0.77 |
| THDCA | -0.15 | 0.59 |
| TLCA | -0.15 | 0.59 |
| HCA | 0.07 | 0.81 |
| GHCA | -0.04 | 0.88 |
| GUDCA | 0.69** | 0.00 |
| GHDCA | 0.00 | 0.99 |
| DCA | -0.03 | 0.93 |
| LCA | -0.23 | 0.41 |
| LCA-3S | 0.18 | 0.52 |
| 7-KetoLCA | 0.03 | 0.92 |
| 12-DHCA | 0.04 | 0.88 |
| 3-DHCA | 0.29 | 0.29 |
| DCA-3S | 0.30 | 0.28 |
